# The effects of vitamin K_2_ on recovery from muscle damaging resistance exercise in young and older adults – The TAKEOVER randomised controlled trial

**DOI:** 10.1249/MSS.0000000000003901

**Published:** 2026-03-05

**Authors:** Hannah Lithgow, Lynsey Johnston, Frederick Ho, Emma Dunning, Shinya Nakada, Carlos Celis-Morales, Truls Raastad, Angus M Hunter, Jennifer S Lees, Patrick B Mark, Terry J Quinn, Stuart R Gray

**Affiliations:** 1 School of Cardiovascular and Metabolic Health, University of Glasgow; 2 School of School of Energy, Geoscience, Infrastructure and Society, Heriot-Watt University; 3 School of Life Sciences, University of Glasgow; 4 School of Health and Wellbeing, University of Glasgow; 5 Human Performance Lab, Education, Physical Activity, and Health Research Unit, Universidad Católica del Maule, Talca, Chile; 6 High-Altitude Medicine Research Centre (CEIMA), Universidad Arturo Prat, Iquique, Chile; 7 Department of Physical Performance, Norwegian School of Sports Science; 8 Department of Sports Science, Nottingham Trent University; 9 Institute of Sports Science and Innovation, Lithuanian Sports University; 10 School of Medicine, Medical Sciences & Nutrition, University of Aberdeen; 11 Faculty of Health Sciences and Sport, University of Stirling

**Keywords:** Resistance exercise, muscle damage, recovery, inflammation, vitamin K2

## Abstract

**Purpose:**

Vitamin K_2_ supplementation has emerged as a strategy to enhance recovery and modulate post-exercise physiological responses. This study aimed to assess the effects of vitamin K_2_ on recovery from muscle-damaging exercise in young and older adults.

**Methods:**

Healthy young (18-40 years) and older (65+ years) adults were randomly assigned to either vitamin K_2_ (menaquinone 7, MK-7, 240 μg/day) or placebo (cellulose) for 12 weeks in this double-blind randomised controlled trial. Before and after supplementation, knee extensor maximal torque, functional ability, muscle soreness, and systemic blood markers of muscle damage and inflammation were measured before (0h) and 3h, 24h, 48h, and 72h post-exercise. Data were analysed using regression and mixed models.

**Results:**

Seventy one participants (35 young and 36 older) completed the study, with 12 weeks of vitamin K_2_ supplementation increasing circulating MK-7 levels (p-value <0.001). There were no supplement*time effects for any variables. Significant supplement*time*older age interaction effects were noted for electromechanical delay (EMD) (p-value = 0.03), electromyography root mean square (RMS) (p-value = 0.01), IL-6 concentrations (p-value <0.001), and creatine kinase (CK) levels (p-values = 0.02). In older adults, after 12 weeks, EMD appeared lower at all time points and RMS higher post exercise in the vitamin K_2_ group. No clear pattern in IL-6 or CK were observed, but at 72h post-exercise CK was lower in older adults in the Vitamin K_2_ group.

**Conclusions:**

Vitamin K_2_ supplementation had no effect on muscle strength, physical function, muscle soreness or inflammatory responses in the recovery period after a bout of resistance exercise. Effects of supplementation were observed on EMD, RMS, IL-6 and CK by age, and warrant further investigation.

**Trial registration:**

Prospectively registered on 21st December 2020, trial registration clinicaltrials.gov ID NCT04676958 https://clinicaltrials.gov/ct2/show/NCT04676958

## Introduction

Physical activity guidelines recommend that muscle strengthening exercises are performed on at least 2 days per week (1), with the consensus that resistance exercise is the most efficacious way to increase muscle mass and strength, alongside broad ranging health benefits including lowering risk of premature mortality (2, 3). This is particularly important in older adults as resistance exercise can reduce injury risk, including risk of falls and fractures, and subsequently promote longevity by supporting healthy ageing (4). Yet, less than half as many people meet the muscle strengthening physical activity guidelines compared to the aerobic recommendations, with participation particularly low in older adults (5). Whilst there are many barriers to muscle strengthening exercise, one contributing factor is that people unaccustomed to exercise commonly experience exercise-induced muscle damage (EIMD) with symptoms of delayed onset muscle soreness (DOMS) up to 14 days after a bout of exercise. The consequences of EIMD include a temporary loss of muscular function, experiencing muscle soreness and pain, reduced muscle force production, and an increase in inflammation (6). This is particularly problematic in an older adults, who are more susceptible to exercise-induced muscle dysfunction (7), which may place individuals at risk of falls (8) and reduce participation in physical activity and exercise in the short-term (9).

For these reasons developing strategies to ameliorate muscle damage responses to resistance exercise are needed, and data indicates that vitamin K_2_ may be effective (10). Vitamin K includes a group of structurally related compounds named phylloquinone (vitamin K_1_) and menaquinone (vitamin K_2_ ; MK-n), which differ in their biological activity and dietary abundance. Long chain menaquinones, such as MK-7, are usually found in animal products and fermented foods and are the most bioactive of the vitamin K family. However, MK-7 concentration in many foods is very low (<10 µg/100 g) and with the western diet containing few fermented foods, intake is likely to be lower than the current daily adequate intake (AI) of 1 µg/kg/day set by the European Food Safety Authority (EFSA) (11). Vitamin K is an essential cofactor for gamma carboxylation, required for the effective function of a range of proteins (12) including those involved in bone remodelling, vascular calcification, glucose handling, inflammation, and neuromuscular function (13–16). Given that these roles are associated with EIMD and recovery after exercise, it is possible that vitamin K_2_ could significantly influence the physiological responses to resistance exercise, although this has yet to be studied.

The primary objective of this study, therefore, was to determine whether 12 weeks of supplementation with vitamin K_2_, specifically MK-7, affects recovery following a bout of resistance exercise in young and older adults. A secondary objective of this study was to determine the effect of supplemental MK-7 on systemic markers of inflammation and to explore if the responses differed by age.

## Methods

### Trial design

The current study was a double blind randomised controlled trial (RCT) with participants randomly assigned to either control or treatment group. Allocation was carried out in a 1:1 ratio, stratified by age (18-40 and 65+ years) and sex, with randomisation in randomly permuted blocks carried out, by an independent colleague using online software (sealedenvelope.com). The trial was registered at clinicialtrials.gov (ID: NCT04676958) and the protocol published (17). In brief, participants attended the laboratory for a screening visit then 4 consecutive days of pre-intervention testing, followed by 12 weeks of supplementation, with the 4 days of testing repeated.

The study was approved by the University of Glasgow Medical, Veterinary, Life Sciences College Ethics Committee [Project No: 200190189] on 10^th^ December 2020 and was conducted in accordance with the ethical standards of the 1964 Declaration of Helsinki. Participants were presented with the participation information sheet that detailed the study aims, protocols, and risks associated with the study. Written informed consent was obtained from the participants following verbal acknowledgement of the study information and an opportunity to ask questions.

### Participants

Participants were recruited to the study from the Glasgow area between June 2021 and February 2023, via poster and newspaper advertisements around the local community. The sample size was chosen to be able to (within age groups) detect a physiologically relevant difference in knee extensor maximal torque, based on data from within our laboratory, of 10% with a SD of 10%, with a power of 80% and alpha level of 0.05. This required a sample size of 17 in each group and so the aim was to recruit 20 participants to each supplement group (separately for young and old age groups) to account for drop out.

### Screening visit

Potential participants were assessed for eligibility based on the inclusion and exclusion criteria: no plans to change lifestyle (activity and nutrition) during the study period; not currently, or in the last year, participating in more than 1h per week of vigorous aerobic physical activity or any resistance exercise; BMI ≤ 30 kg/m^2^; not have diabetes mellitus, severe cardiovascular disease, dementia, seizure disorders, liver disease, uncontrolled hypertension (>150/90 mmHg at baseline measurement), cancer or cancer that has been in remission <5 years; not have ambulatory impairments which would limit ability to perform assessments of muscle function; not currently taking Vitamin K_2_ supplements; not currently taking Vitamin K antagonists/anticoagulants (e.g. warfarin); not be a current smoker; not have a history of drug abuse; not be currently taking medication known to affect muscle (e.g. steroids). Participants were then asked to complete a Food Frequency Questionnaire to assess habitual diet and asked to maintain their habitual diet throughout involvement in the study. Participants were then issued with an accelerometer (GeneActiv) to wear for 7 days to determine habitual physical activity levels. One-repetition maximum (1RM) was determined for two exercises: bilateral leg-extension and chest press on fixed-weight machines. The exercise was first demonstrated, then the participant was seated and the equipment adjusted. Participants performed 1 repetition of the exercise without weight, then weight was applied in increments to the point of failure.

### Protocol

Participants attended the laboratory for 4 consecutive days pre- and then post-supplementation, with participants instructed to maintain their habitual activity and diet for the entire intervention. Participants arrived at the laboratory (on the first of the 4 consecutive days) between 07:00-08:30 in a fasted state (10 h) for baseline data collection (0h timepoint) and the following outcomes were measured body composition, grip strength, vastus lateralis muscle thickness, five times sit-to-stand time, muscle soreness and muscle function, and a blood sample was collected. Participants then performed the resistance exercise protocol to induce EIMD.

The exercise involved bilateral leg extension exercises at 70% of one-repetition maximum (1RM), with 5 sets of 15 repetitions performed. Each repetition was performed for 4s – 2s in the concentric and 2s in the eccentric phase, with a 90 second passive rest between sets. Where necessary, the concentric phase was assisted. Following this the same protocol was performed for chest press. Participants were then offered a standardised snack. Grip strength, vastus lateralis muscle thickness, five times sit-to-stand time, muscle soreness and muscle function, and a blood sample were collected 3, 24, 48 and 72 hours post exercise.

Study supplements were then dispensed to participants. The control group received the placebo, which was one 380mg micro-crystalline cellulose tablet per day. Participants in the treatment group received one 380mg micro-crystalline cellulose tablet containing 240 μg vitamin K_2_ (MK-7) (K2VITAL™ 0.2% DELTA powder) per day for 12 weeks. Participants were asked to take the supplement with lunch or dinner due to the fat-soluble properties of vitamin K_2_. The control and vitamin K_2_ capsules were identical in look and taste, and both were provided by Kappa Bioscience AS (Oslo, Norway). The manufacturer had no role in the data collection or analysis. Adherence to the supplementation intervention was established by counting the number of tablets remaining in the container when the participant returned for post-supplementation testing.

Following the 12 week intervention period the outcome collection and EIMD protocol were repeated as it was pre-supplementation.

### Outcome measurements

#### Knee extensor muscle strength

Knee extensor muscle maximal torque was measured in the right leg during an isometric maximal voluntary contraction (MVC) with participants secured in a dynamometer, with a knee angle of 90º and force recorded throughout via a load cell (Biometrics Ltd, Newport, UK). A cuff was placed proximal to the ankle with the strain gauge attached to the cuff and the back of the dynamometer chair. A minimum of three contractions (for ~5 s, with 3 min rest between contractions) were conducted and the highest value was used in the subsequent analysis. Peak torque was calculated from peak force and limb lever length (distance from lateral condyle of the femur to the midpoint of the cuff). The rate of torque development (RTD) was calculated from the MVC data. The torque at time instants 0, 50, 100, 200 and 300 ms was determined and the RTD for each time interval (i.e. RTD50, RTD100, RTD200 and RTD300) calculated by subtracting from the torque at each time point the torque at 0 ms and dividing by the time interval (18).

#### Neuromuscular function

During the measurement of knee extensor muscle strength, a surface electromyography (sEMG) electrode was positioned on the vastus lateralis muscle, in accordance with SENIAM guidelines (19), and recorded during each MVC. To reduce the skin impedance, the skin was cleaned with isopropyl alcohol. sEMG signals were root mean square (RMS) processed, with average RMS calculated over a 500 ms period, 250 ms each side of peak force. Electromechanical delay (EMD) was determined as the time between the start of electrical activation (sEMG signal) and the start of mechanical activation (onset of force production) in the vastus lateralis muscle.

#### Body composition

Bioelectrical impedance analysis (BIA) was used to measure percentage body fat (Tanita TBF-300, Tokyo, Japan).

#### Sit-to-Stand Test

Physical functioning was measured using the Five Times Sit-to-Stand Test, which is detailed elsewhere (20). Participants were familiarised with the protocol, then asked to perform the test twice, separated by a 2 minute rest.

#### Muscle soreness

With participants in a supine position, soreness was assessed using a muscle soreness questionnaire for soreness in the hamstrings, vastus medialis, rectus femoris, and vastus lateralis. Soreness was determined on a 1 to 10 rating scale, with 1 as ‘Normal’ and 10 as ‘Very Very Sore’. Pressure was applied by the researchers’ index and middle fingers at the proximal and distal ends of the muscle and a rating obtained. The ratings were summated for each muscle, then subsequently totalled for a rating of overall muscle soreness.

#### Blood sample collection and analysis

All blood samples were collected via venepuncture from an antecubital vein with the participant seated on a laboratory bed. For plasma, whole blood was centrifuged at 1500 rpm for 15 min at 4 °C within 30 min of collection. For serum, whole blood was incubated at room temperature for 30 min prior to centrifugation at 1000 x g for 10 min at 4 °C. The resulting plasma and serum were aliquoted into 1.5 ml vials and stored at minus 80 °C for subsequent analysis.

Total cholesterol, HDL-cholesterol, LDL-cholesterol, triglycerides, plasma glucose, and creatine kinase (CK; timepoints 0h-72h) were measured on a Cobas c311 analyser (Roche Diagnostics, Burgess Hill, UK), and plasma insulin on a Cobas e411 analyser (Roche Diagnostics, Burgess Hill, UK). Plasma 25-OH vitamin D3 (25(OH)D) levels were measured in EDTA plasma using a validated liquid chromatography-tandem mass spectrometry (LC-MS/MS) as previously described (21). Vitamin D deficiency was defined as a 25(OH)D concentration <25 nmol/L, insufficiency between 25 and 50 nmol/L, and sufficiency ≥50 nmol/L.

Plasma dephosphorylated-uncarboxylated matrix gla-protein (dp-ucMGP) levels were determined using the commercially available CE-marked IVD chemiluminescent InaKif MGP assay on the IDS-iSYS system (IDS, Boldon, UK) as previously described (22). The within-run and total modulation of this assay were 0.8–6.2% and 3.0–8.2%, respectively. The assay measuring range was between 200–12,000 pmol/L and linear up to 11,651 pmol/L. Dp-ucMGP levels <300 pmol/L are in the normal healthy range and levels >500 pmol/L reflect vitamin K insufficiency (23). Total matrix gla-protein (tot-MGP) levels were determined by an enzyme-linked immunosorbent assay (ELISA) kit (Reddot Biotech Inc., Kelowna, Canada). Serum Gla-type Osteocalcin (Gla-OC), undercarboxylated Osteocalcin (Glu-OC) levels were determined by an enzyme immunoassay (EIA) kits (Takara Bio Inc., Shiga, Japan).

Serum concentrations of menaquinone-7 (MK-7) were measured by the contract laboratory Vitas AS (Oslo) by a validated method (internal report, Vitas AS) as detailed previously (24). Briefly, serum samples were thawed and mixed. Then, serum samples, calibration standards, and quality control samples (80 μL) were combined with 300 μL of isopropanol containing 18O MK-7 (10 ng/mL) as an internal standard. The samples were shaken at 1350 rpm for 6 min to precipitate proteins. The isopropanol layer containing MK-7 was separated from the precipitated proteins and analysed by liquid chromatographytandem mass spectrometry (LC-MS/MS). Chromolith ® SpeedROD RP-18e (4.6 mm × 50 mm, Merck) was used as the analytical column and Chromolith RP-18e (4.6 mm × 10 mm, Merck) as the guard column. The injection volume was 50 μL.

Systemic inflammation was measured at timepoints 0h, 3h, and 24h via a cytokine panel (Tumour Necrosis Factor alpha (TNF-alpha), interleukin (IL)-6, IL-1Ra, IL-10, IL-1a) analysed using an Ella Automated Immunoassay System (ProteinSimple, Bio-Techne, Minneapolis, USA). All assays were calibrated and quality controlled using the manufacturer’s reagents.

### Statistical analysis

Data and statistical analysis were performed by the trial statistician who was blinded to the group assignment/randomisation. The mean (SD) of outcomes for intervention and control groups were calculated separately. The effect of the intervention accounting for baseline measurements were estimated using linear regression models. Supplement (intervention vs. control), Time (pre- vs. post-intervention), and Supplement x Time were included as predictors, as well as prognostic factors (age, sex, country of origin). Supplement x Time variables were the primary predictor of interest indicating intervention effectiveness. Prognostic factors were included to increase statistical power. To examine if there was any subgroup difference by age group, analysis was performed to examine the interaction of Age Group x Supplement x Time in the same linear regression models. Because outcomes were measured over a time course both before and after intervention, analysis was also conducted to examine if there were any changes to the time trend in the intervention group post-intervention. This was done using a linear mixed model instead of linear model because there were multiple measurements from the same participants. Participants identifier was used as random intercept. The time trend difference was estimated via the interaction of Time in hour x TVE, where TVE is a binary variable coded as 0 = pre-intervention of both groups or post-intervention of control group; 1 = post-intervention of intervention group. This provides the maximum statistical power assuming the same time trend among control group before and after intervention and the intervention group before intervention. Data were compared between groups at each time point, pre and post supplementation, via t-test with false discovery rate (FDR) correction and effect sizes for differences presented.

## Results

### Participants and physical characteristics

**Figure 1:**
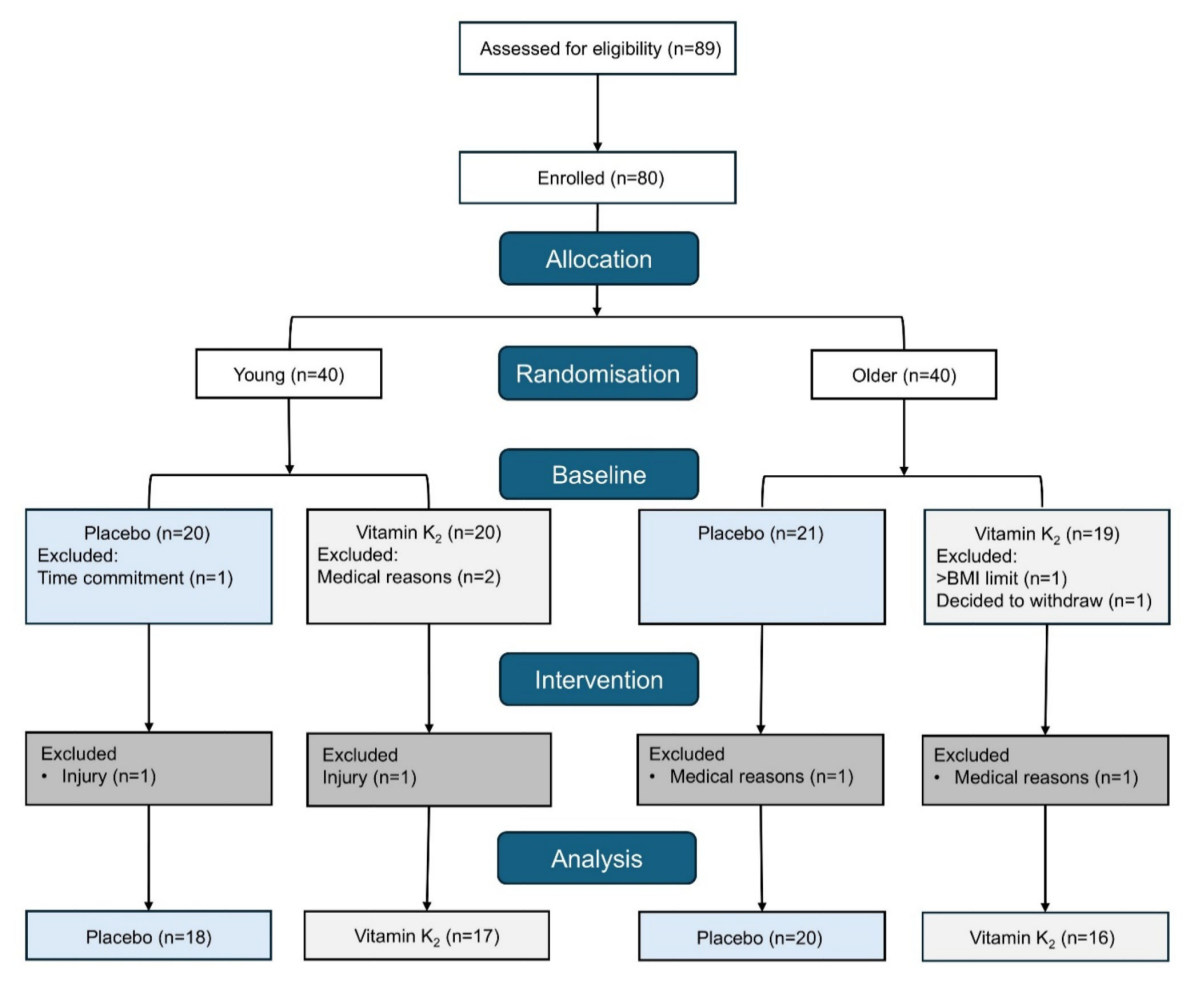
Consort diagram for the TAKEOVER Trial.

Eighty participants were enrolled in the study, with 75 completing baseline data collection, and 71 completing post-supplementation testing (Figure 1). One young male participant did not return for the post-supplementation testing due to injury (unrelated to the study); one young male participant did not complete all timepoints of the post-supplementation testing due to injury (unrelated to the study); two older male participants did not return for post-supplementation testing due to medical diagnoses (unrelated to the study). There was 98.4% compliance with the supplementation intervention for the 12 weeks (5950 tablets taken out of 6048 total). Baseline physical characteristics are presented stratified by age and intervention group (Table 1).

Data presented as means±SD. * n=69 due to accelerometer not worn by two participants during the night.

### Vitamin K_2_ concentration and markers of K_2_ activity

**Figure 2:**
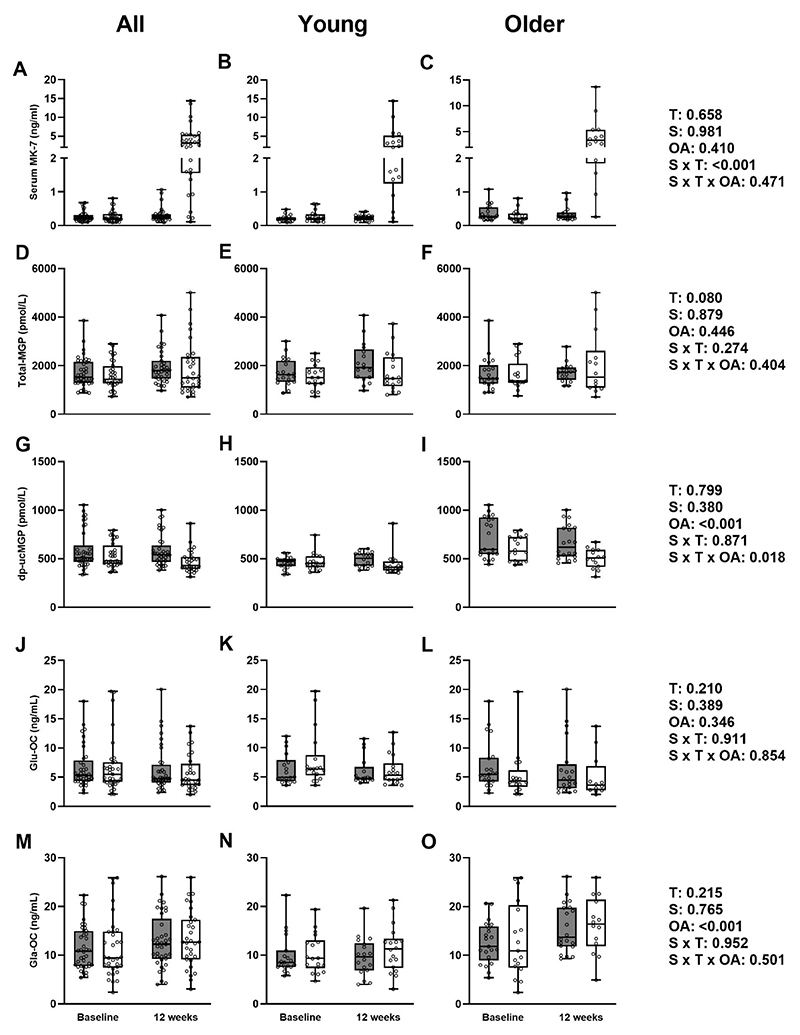
Mean (±SEM) serum MK-7 (A, B, C), plasma dp-ucMGP (D, E, F), plasma total-MGP (G, H, I), plasma Glu-type osteocalcin (J, K, L), and Gla-type uc osteocalcin (M, N, O) in placebo (◼) and vitamin K_2_ (□) groups in all, young, and older adults. T: time effect; S: supplement effect; OA: older age effect.

A significant supplement*time interaction was observed for MK-7 concentration (Figure 2, A-C). MK-7 concentration was significantly higher after 12 weeks in the vitamin K_2_ group, compared to placebo in both young and older adults. There was no effect of older age on MK-7 concentrations. There was no interaction or effect of supplement, time, or older age for plasma total MGP (Figure 2, D-F). However, there was an effect of older age on plasma dp-ucMGP with levels higher in older, compared to younger adults (Figure 2, G-I). A supplement*time*older age interaction was also observed with older, but not younger, adults exhibiting lower uncarboxylated MGP at 12 weeks in the vitamin K_2_ group. For Glu-OC and Gla-OC, there was an effect of older age on Gla-OC with higher concentrations reported in older adults compared to their young counterparts (Figure 2, M-O), but no main or interaction effects on Glu-OC (Figure 2, J-L).

### Muscle function

**Figure 3:**
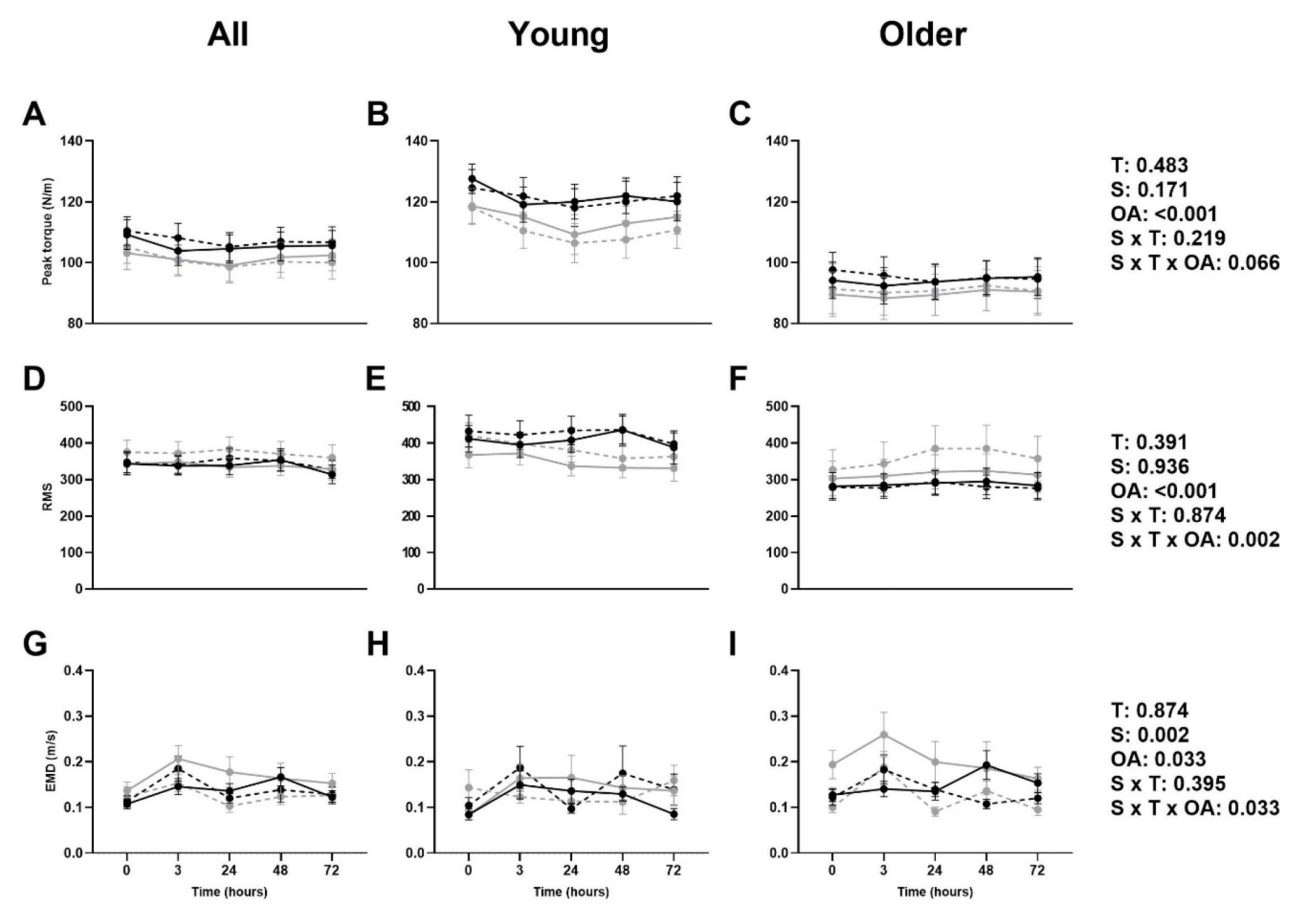
Mean (±SEM) peak torque (A, B, C), EMG root-mean-square (RMS; D, E, F), and electromechanical delay (EMD; G, H, I) at baseline (▬) and 12 weeks (---) in placebo (•) and vitamin K_2_ (○) groups in all, young, and older adults. T: time effect; S: supplement effect; OA: older age effect.

There was no effect of time, or supplement*time interactions on either peak torque or EMD, however there was an effect of older age. Older adults exhibited lower peak torque, lower RMS-processed sEMG signals, and higher EMD. No supplement or supplement*time*older age interactions were observed for peak torque. There was a significant supplement effect and supplement*time*older age interaction on RMS, with RMS higher post-exercise in older adults after the 12 week vitamin K_2_ supplementation intervention (Figure 3, F).

Regarding EMD, there was a significant supplement effect and supplement*time*older age interaction, with EMD reduced in the older, but not younger, adults in the vitamin K_2_ group at 12 weeks (Figure 3, I). No significant time trends were seen in peak torque (p=0.334), RMS (p=0.328), or EMD (p=0.105).

### Muscle damage and Sit-to-Stand time

**Figure 4:**
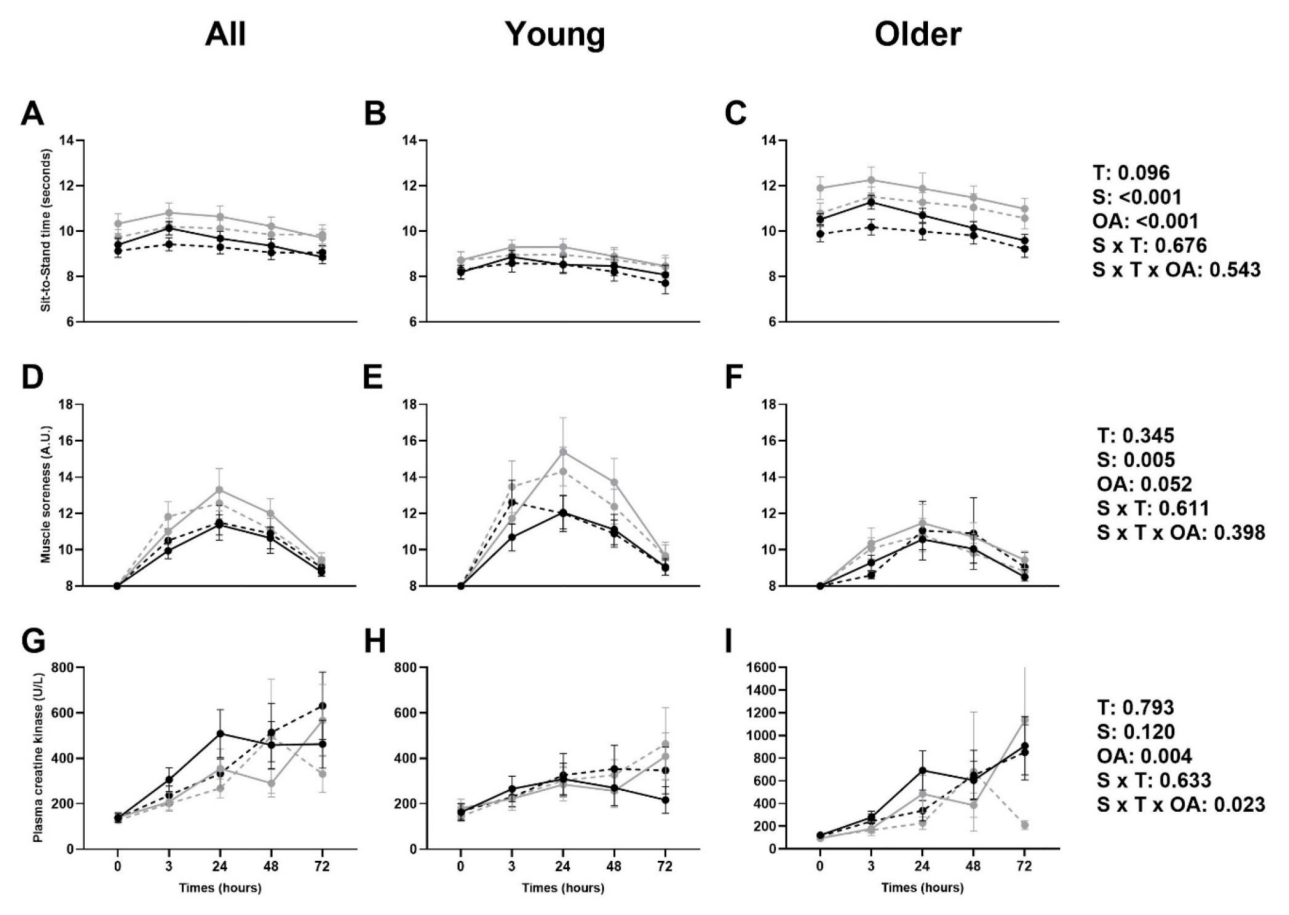
Mean (±SEM) physical functioning (five time sit-to-stand test time; A, B, C), muscle soreness (D, E, F), and plasma creatine kinase (G, H, I) at baseline (▬) and 12 weeks (---) in placebo (•) and vitamin K_2_ (○) groups in all, young, and older adults. T: time effect; S: supplement effect; OA: older age effect.

There were effects of supplement and older age on the Sit-to-Stand Test, with older adults taking longer to complete the test and a slower time reported in the vitamin K_2_ groups (Figure 4, A-C). No time, supplement*time or supplement*time*older age interactions were reported. There was an effect of supplement on muscle soreness with less soreness reported in the vitamin K_2_ group. No time, older age, supplement*time or supplement*time*older age interactions were reported (Figure 4, D-F). There was an effect of age, indicating higher concentrations in the older adult cohort, and a supplement*time*older age interaction effect for creatine kinase (Figure 4, G-I). No clear patterns in CK were observed, although CK levels appear lower 72h post-exercise in older adults in the Vitamin K_2_ group. No time, supplement or supplement*time interaction effects were reported. No significant time trends were seen in sit-to-stand time (p=0.569), muscle soreness (p=0.628) or creatine kinase (p=0.101).

### Cytokine response

**Figure 5:**
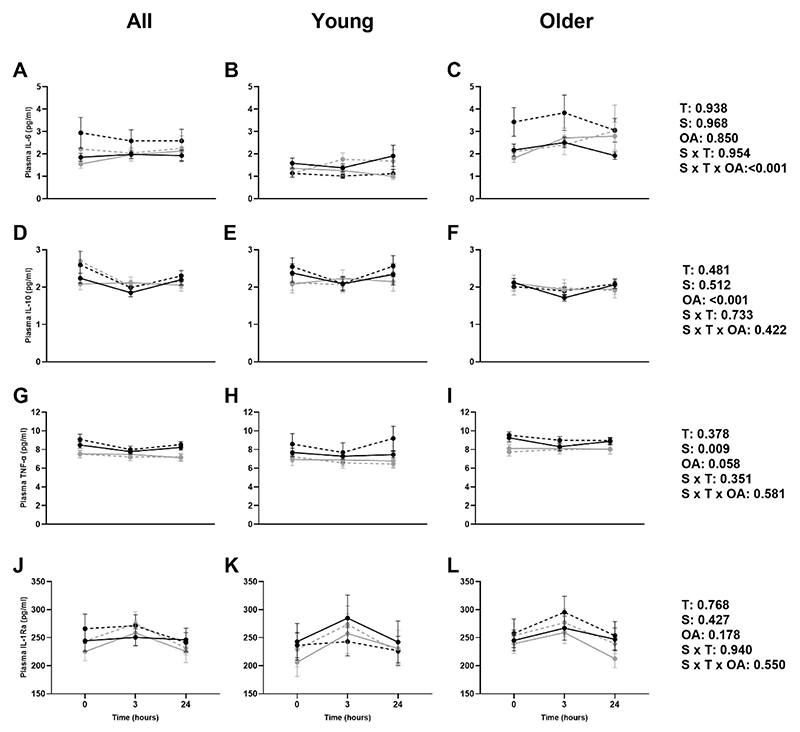
Mean (±SEM) plasma interleukin-6 (IL-6; A, B, C), plasma interleukin-10 (IL-10; D, E, F), plasma tumour necrosis factor alpha (TNF-α; G, H, I), and plasma interleukin-1 receptor antagonist (IL-1Ra; J, K, L) at baseline (▬) and 12 weeks (---) in placebo (•) and vitamin K_2_ (○) groups in all, young, and older adults. T: time effect; S: supplement effect; OA: older age effect.

A significant supplement*time*older age interaction was found for IL-6 (Figure 5, A-C), with no time, supplement, older age or time*supplement interaction effects observed. In older adults in the placebo group IL-6 levels appeared higher at all time points after the 12 weeks. There was an effect of older age for IL-10 concentration, with older adults generally displaying lower levels, with no time, supplement, time*supplement or time*supplement*older age interaction effects observed (Figure 5, D-F). There was an effect of the supplementation on TNF-α, with the placebo group having higher levels compared to the vitamin K_2_ group, with no time, older age, time*supplement or time*supplement*older age interaction effects observed (Figure 5, G-I). Analysis revealed no effects on IL-1Ra (Figure 5, J-L). No significant time trends were seen in IL-6 (p=0.515), IL-10 (p=0.954), TNF-α (p=0.431) or IL-1Ra (p=0.977).

## Discussion

The current study investigated the effects of 12 weeks of vitamin K_2_ (MK-7) supplementation on muscle recovery following resistance exercise in young and older adults. On top of this, systemic markers of inflammation were assessed, and potential age-related differences in response were explored. Our findings showed that although the supplementation protocol increased circulating MK-7 concentrations, with a corresponding reduction in uncarboxylated matrix gla-protein (dp-ucMGP) only in older adults, there was no clear effect on muscle damage, muscle soreness, physical function, or muscle strength recovery post-exercise. The subgroup analysis did indicate some differential responses by age group which require further investigation.

Supplementation with 240 μg vitamin K_2_ (MK-7) per day for 12 weeks led to a significant increase in MK-7 concentration in both younger and older adults. These data highlights that this dose is tolerable and successful in raising vitamin K status. It is interesting to note that older adult’s systemic concentration of MK-7 increased to a similar extent as in younger adults, speculating that there is no effect of ageing on absorption of the vitamin in this form. This increase in MK-7 can be associated with heightened activity of vitamin K_2_. Due to the vitamin K-dependant carboxylation of specific proteins, the uncarboxylated forms (dp-ucMGP, ucOC) are considered biomarkers of vitamin K status and activity (25). Whilst the current study found an increase in MK-7, there was no effect on ucOC, but there was a decrease in dp-ucMGP following vitamin K_2_ supplementation in older, but not younger, adults. This is in contrast to previous work with vitamin K_1_, where supplementation for 25 days (500µg/day) resulted a similar response in younger and older adults in circulating vitamin K_1_, percentage ucOC, and a protein induced in vitamin K absence or antagonism – Factor II (PIVKA-II) (26). The differential age responses in the current study may reflect the higher levels of dp-ucMGP in older adults at baseline with more scope, therefore, for a reduction to occur.

The primary outcome in the current study was knee extensor maximal torque and in spite of the increases in circulating MK-7 levels, there was no effect of supplementation with vitamin K_2_ on torque recovery post-exercise in either young or older people. This is also reflected in the more functional test of sit-to-stand time, where no effect of vitamin K_2_ supplementation was observed. Previous work in this area is scarce, but previous cross-sectional work investigating vitamin K_2_ and muscle function has mixed results. A lower vitamin K status has previously been found to be associated with low grip strength, poorer physical function, frailty risk and falls related hospitalisation (27–29). However, analysis of data from older adults found no association of vitamin K_2_ intake with grip strength, time-up-and-go performance or risk of falls, although associations were observed for vitamin K_1_ (30). In analysis of the U.S. National Health and Nutrition Examination Survey data set it was shown that dietary vitamin K intake was positively associated with skeletal muscle mass, in males but not females, and grip strength, at low but not high intakes (31). In RCTs, vitamin K_2_ supplementation has been shown to have no effect on postural sway, falls or physical function (short physical performance battery, Berg balance scale, time-up-and-go performance) in older adults (32) with no effects on grip strength or short performance physical battery test performance in older adults with vascular disease (33).

The current study is the first to investigate the effects of vitamin K_2_ supplementation on muscle strength and physical function recovery after a bout of resistance exercise and indicates, similarly, no evidence of effect. At this point it is worth noting that to enhance external validity the current study used standard resistance exercise to induce EIMD, and it remains possible, although we would contend unlikely, that more severe EIMD protocols that result in greater muscle damage and dysfunction may be more amendable to modulation from vitamin K_2_. In other areas of research, such as chronic kidney disease (CKD), it appears that although vitamin K_2_ supplementation has little effect on vascular health there appears to be benefits when supplementation is with vitamin K_1_ (34, 35), and this is worth exploring for its potential to influence post-exercise recovery (36, 37). On top of this, although there is debate about levels of dp-ucMGP to establish clinically meaningful deficiency, the majority of the participants included in the current study were not obviously vitamin K deficient and whether benefits may be realised in a similar study of participants recruited based on being vitamin K deficient remains to be established.

However, our study did find a significant elevation in RMS and a reduction in EMD in the older adult group supplemented with vitamin K_2_, which may suggest an improvement in neuromuscular efficiency. Interestingly, the reduction in EMD appears to be independent of the bout of resistance exercise with lower values seen at 12 weeks at all time points. RMS, on the other hand was similar pre-exercise but elevated at all time points following the bout of exercise (3h-72h). Given that vitamin K-dependent proteins such as Gas6 and periostin are implicated in neuromuscular signalling and myocyte survival (38), it is possible that vitamin K_2_ supplementation facilitated faster neuromuscular transmission in the older cohort. This requires to be confirmed in further appropriately designed trials.

As well as a loss of function, EIMD is characterised by a transient soreness, and elevated biomarkers of muscle damage, such as CK (6). The current study found no effect of vitamin K_2_ supplementation on muscle soreness, which is also reflected in the lack of any clear effect on inflammatory responses to exercise, with inflammation being involved in the soreness response (39, 40). The current data did reveal a supplement*time*old age interaction effect for CK, although the trend analysis found no differences between groups at 12 weeks. Data do appear to show that in older adults, vitamin K_2_ supplementation may result in a decrease in CK at 72 hours post-exercise, although as with other observations this finding requires further exploration.

A strength of the current study was the robust double-blind, RCT design, which minimised bias and ensured high methodological rigor. Additionally, stratification by age allowed for a comprehensive analysis of age-related differences in response to vitamin K_2_ supplementation. However, this study also has limitations. First, the sample size, while sufficient to detect physiologically relevant changes in knee extensor torque, may have limited our ability to detect smaller but potentially meaningful effects on muscle recovery and inflammation. Moreover, the reduction in torque following the muscle-damaging protocol was ≤7%, which is lower than both the predicted value and the amount used for the sample size calculation. Second, dietary vitamin K intake was not tightly controlled beyond exclusion of participants taking vitamin K_2_ supplements, which may have influenced baseline vitamin K status. Thirdly, whilst this was the first study in this area it is possible that a higher dose of vitamin K_2_ or longer duration of supplementation is required to observe any effect, and further work should consider a dose-response investigation. Fourthly, although we compared young and older people, the older adults included in this study were generally very healthy and whether results may differ if we included a sample of older adults with frailty/sarcopenia, although recruitment of such participants to exercise trials is a challenge (41). Last but not least, the study is not powered to correct for type 1 error due to multiple testing. Given the number of tests conducted, significant findings from this study should be replicated in future work.

In conclusion, our findings demonstrate that in a sample of young and older adults, 12 weeks of vitamin K_2_ (MK-7) supplementation significantly improved vitamin K status but did not enhance muscle recovery or reduce systemic inflammation following an acute bout of resistance exercise. These results suggest that while vitamin K_2_ is important for overall health, its role in exercise recovery may be limited, at least within the parameters of this study. Interestingly, exploratory findings indicate an increase in RMS and decrease in EMD and CK, with less clear effects in IL-6, in older adults, following vitamin K_2_ supplementation, and further work is required to explore this in more depth.

## Figures and Tables

**Table 1:** Anthropometric characteristics and physical activity levels of the placebo and vitamin K_2_ group (n=71).

	All		Young		Older	
	Placebo	Vitamin K_2_	Placebo	Vitamin K_2_	Placebo	Vitamin K_2_
N (male:female)	38 (20:18)	33 (14:19)	18 (9:9)	17 (7:10)	20 (11:9)	16 (7:9)
Age (y)	51 ± 23	49 ± 23	28 ± 6	28 ± 5	72 ± 5	72 ± 5
Body mass (kg)	71.2 ± 12.7	69.3 ± 12.9	72.3 ± 11.0	69.0 ± 14.1	70.2 ± 14.3	69.7 ± 11.9
BMI (kg/m^2^)	24.6 ± 3.3	24.0 ± 3.1	24.4 ± 2.7	24.2 ± 3.3	24.9 ± 3.9	23.8 ± 3.0
Body fat (%)	26.3 ± 7.6	25.7 ± 7.0	24.4 ± 7.4	22.8 ± 5.9	28.1 ± 7.6	28.8 ± 6.8
Sleep duration (min/d)*	425 ± 61	440 ± 50	411 ± 61	418 ± 33	438 ± 60	461 ± 55
Sleep efficiency (%)*	72 ± 9	73 ± 7	72 ± 9	72 ± 6	72 ± 9	74 ± 8
Inactivity (min/day)	505 ± 82	506 ± 70	535 ± 71	502 ± 79	479 ± 83	510 ± 60
Light intensity (min/d)*	204 ± 67	192 ± 60	188 ± 60	180 ± 56	218 ± 72	204 ± 63
Moderate to vigorous intensity exercise (min/d)*	118 ± 53	110 ± 62	120 ± 46	114 ± 56	102 ± 42	85 ± 36

Data presented as means±SD.

* n=69 due to accelerometer not worn by two participants during the night.
